# The Role of Diet and Nutrition in Cancer Development and Management: From Molecular Mechanisms to Personalized Interventions

**DOI:** 10.3390/foods14101788

**Published:** 2025-05-18

**Authors:** Maxim Ruban, Elizaveta Pozhidaeva, Larisa Bolotina, Andrey Kaprin

**Affiliations:** 1 P. Hertsen Moscow Oncology Research Institute—Branch of the National Medical Research Radiological Centre, 105425 Moscow, Russia; 2 Department of Urology and Operative Nephrology, Peoples’ Friendship University of Russia (RUDN University), Mikluho-Maklaya St., 6, 117198 Moscow, Russia

**Keywords:** cancer, diet, nutrition, nutrients, microbiota

## Abstract

Diet plays a crucial role in cancer development and progression, beyond traditional risk factors. This review aims to summarize current evidence on the role of diet and specific nutrients in cancer development and progression, focusing on molecular mechanisms. We also discuss the potential of personalized dietary interventions, based on tumor and patient characteristics, in enhancing cancer prevention and treatment strategies. The review covers the impact of calories, protein, sugar, and other dietary components on signaling pathways and growth factors involved in carcinogenesis. We examine the influence of obesity, insulin resistance, and other metabolic factors on cancer risk and outcomes. The article also explores current dietary strategies, including calorie restriction, ketogenic diets, and the role of the gut microbiome in modulating response to anticancer therapies. Finally, we highlight the need for further research to develop targeted, personalized dietary recommendations based on an individual’s tumor profile, stage of disease, and other clinical factors. Integrating such personalized dietary approaches into cancer prevention and treatment holds promise for improving patient outcomes and quality of life.

## 1. Introduction

Cancer is a leading cause of death worldwide, and its incidence continues to rise. While genetic factors and environmental exposures are well-established risk factors, the role of diet in cancer development and progression has gained increasing attention in recent years. Accumulating evidence suggests that dietary habits and specific nutrients can modulate cancer risk and influence treatment outcomes, highlighting the importance of considering dietary factors in cancer prevention and management [1,2].

The relationship between diet and cancer is complex, involving multiple biological pathways and mechanisms. Nutrients and bioactive compounds in food can influence carcinogenesis by modulating inflammation, oxidative stress, DNA repair, cell proliferation, and apoptosis [3,4]. Moreover, dietary factors can interact with the gut microbiome, which has emerged as a key player in cancer development and response to therapy [5].

Despite the growing recognition of diet’s role in cancer, translating this knowledge into personalized dietary recommendations remains a challenge. Individual responses to dietary interventions can vary widely, depending on factors such as genetic background, tumor characteristics, and stage of disease [6,7]. Therefore, a deeper understanding of the molecular mechanisms underlying the diet–cancer relationship and the development of targeted dietary strategies based on individual patient profiles are crucial for improving cancer prevention and treatment outcomes [8].

In this review, we aim to summarize current evidence on the role of diet and specific nutrients in cancer development and progression, focusing on molecular mechanisms. We also discuss the potential of personalized dietary interventions, based on tumor and patient characteristics, in enhancing cancer prevention and treatment strategies. By integrating the latest findings on the complex interplay between diet, metabolism, and cancer biology, we hope to provide insights into the development of more effective, personalized dietary approaches for cancer prevention and management. Recognizing the complexity of the role of diet in cancer and its variability across cancer types, this review focuses on the general molecular mechanisms underlying these interactions, illustrated with examples from several common cancers.

## 2. Interrelation of Intracellular Mechanisms of Oncogenesis and Nutrition

The development of cancer is a complex, multistep process that involves the dysregulation of various intracellular mechanisms. Accumulating evidence suggests that dietary factors can significantly influence these mechanisms, either promoting or suppressing oncogenesis (Table 1) [7,9]. By understanding the interplay between nutrition and the key intracellular processes involved in cancer development, we can better appreciate the potential of dietary interventions in cancer prevention and treatment.

One of the hallmarks of cancer is chronic inflammation, which creates a microenvironment conducive to tumor growth and progression. Dietary factors can modulate inflammatory pathways, with omega-3 fatty acids exerting anti-inflammatory effects, while refined carbohydrates and saturated fats promote inflammation [10,11]. Similarly, oxidative stress, resulting from an imbalance between pro-oxidants and antioxidants, can lead to DNA damage and mutations that drive carcinogenesis. Antioxidant-rich foods, such as fruits and vegetables, can help mitigate oxidative stress, while pro-oxidants like heterocyclic amines found in charred meat may increase cancer risk [12,13].

Insulin and insulin-like growth factor-1 (IGF-1) signaling pathways also play a crucial role in cancer development as their overactivation can promote cell proliferation and survival. High glycemic load diets, which rapidly raise blood glucose and insulin levels, have been associated with increased cancer risk, while calorie restriction has been shown to reduce insulin and IGF-1 levels, potentially slowing tumor growth [6,14].

Dysregulation of cell cycle checkpoints and evasion of apoptosis are other key mechanisms that allow cancer cells to proliferate uncontrollably and evade programmed cell death. Nutrients such as folate and vitamin B12 are essential for proper DNA synthesis and repair, helping maintain cell cycle control. Phytochemicals like curcumin and resveratrol have also been shown to induce apoptosis in cancer cells, while saturated fats may inhibit apoptotic pathways [8,15].

In the following subsections, we will delve deeper into each of these intracellular mechanisms, examining the current evidence on how specific dietary factors influence oncogenesis at the molecular level. By gaining a comprehensive understanding of the interrelation between nutrition and cancer biology, we can pave the way for the development of targeted dietary strategies for cancer prevention and treatment.

### 2.1. Inflammation

Chronic inflammation is a hallmark of cancer and plays a pivotal role in tumor initiation, progression, and metastasis. Inflammatory cells, such as macrophages, neutrophils, and lymphocytes, infiltrate the tumor microenvironment and secrete pro-inflammatory cytokines, including interleukin-6 (IL-6) and tumor necrosis factor-alpha (TNF-α). These cytokines activate transcription factors such as nuclear factor-kappa B (NF-κB) and signal transducer and activator of transcription 3 (STAT3), which regulate the expression of genes involved in cell survival, proliferation, and angiogenesis [16,17]. The activation of these pathways creates a microenvironment conducive to tumor growth while simultaneously promoting immune evasion. Chronic inflammation also generates reactive oxygen species (ROS) and reactive nitrogen species (RNS), which induce oxidative stress and cause DNA damage. This damage can lead to mutations in tumor suppressor genes, such as TP53, and oncogenes, such as KRAS, further driving carcinogenesis [18]. The persistent presence of inflammatory mediators also facilitates angiogenesis by upregulating vascular endothelial growth factor (VEGF), which supplies the growing tumor with nutrients and oxygen, thereby enhancing its progression [19].

Dietary factors play a critical role in modulating inflammatory pathways, either exacerbating or mitigating chronic inflammation. Omega-3 polyunsaturated fatty acids (PUFAs), particularly eicosapentaenoic acid (EPA) and docosahexaenoic acid (DHA), exhibit potent anti-inflammatory effects. These fatty acids inhibit the production of pro-inflammatory eicosanoids, such as prostaglandin E2, and reduce the expression of inflammatory mediators by suppressing NF-κB and STAT3 signaling [20,21]. In contrast, diets high in refined carbohydrates and saturated fats promote inflammation by activating NF-κB and increasing circulating levels of pro-inflammatory cytokines like IL-6 and TNF-α [7,22]. High-fat diets also elevate free fatty acid levels, which activate toll-like receptor 4 (TLR4) signaling, further amplifying inflammation in the tumor microenvironment [23].

Chronic inflammation is a critical driver of cancer development, and dietary interventions represent a promising strategy to modulate inflammatory pathways and reduce cancer risk. By targeting key inflammatory mediators and signaling pathways, such as NF-κB and STAT3, dietary factors can influence the tumor microenvironment and potentially mitigate cancer progression. Future research should focus on personalized dietary interventions tailored to an individual’s inflammatory profile and tumor characteristics, which could provide more effective strategies for cancer prevention and management.

### 2.2. Oxidative Stress

Oxidative stress arises from an imbalance between the production of reactive oxygen species and the body’s ability to neutralize them through antioxidant defenses. ROS, which include superoxide anion, hydrogen peroxide, and hydroxyl radicals, are natural byproducts of cellular metabolism and play a role in normal cell signaling and homeostasis. However, excessive ROS levels can cause oxidative damage to cellular macromolecules, including DNA, proteins, and lipids, leading to genomic instability and tumorigenesis. In the context of cancer, oxidative stress contributes to all stages of tumor development, including initiation, promotion, and progression. During the initiation phase, ROS induce DNA damage, such as base modifications, strand breaks, and the formation of mutagenic lesions like 8-oxo-2′-deoxyguanosine, which can result in mutations in critical genes, including tumor suppressors like TP53 and oncogenes like KRAS. These mutations drive the transformation of normal cells into malignant ones, setting the stage for carcinogenesis [24,25].

Beyond initiation, oxidative stress plays a critical role in tumor promotion and progression by creating a microenvironment conducive to cancer cell survival and proliferation. ROS activate redox-sensitive transcription factors, such as nuclear factor-kappa B (NF-κB) and hypoxia-inducible factor-1 alpha (HIF-1α), which regulate the expression of genes involved in cell proliferation, angiogenesis, and survival. For instance, HIF-1α activation under oxidative stress conditions promotes the secretion of vascular endothelial growth factor, facilitating angiogenesis and ensuring an adequate supply of oxygen and nutrients to the growing tumor. Additionally, oxidative stress can suppress the immune response by impairing the function of natural killer (NK) cells and cytotoxic T cells, allowing cancer cells to evade immune surveillance and metastasize to distant organs [26,27].

While oxidative stress is a key driver of tumorigenesis, cancer cells often exploit this phenomenon to their advantage. Tumor cells adapt to high ROS levels by upregulating antioxidant systems, such as glutathione and NADPH production, to maintain redox balance and protect themselves from oxidative damage. This dual role of oxidative stress in cancer highlights its complexity as ROS can both promote tumor progression and induce cancer cell death when levels exceed a critical threshold. This paradox underscores the importance of understanding the context-dependent effects of oxidative stress in cancer biology [28].

Antioxidants, which scavenge ROS and enhance the body’s endogenous antioxidant defenses, have been proposed as a strategy to mitigate oxidative stress and reduce cancer risk. These compounds, which are abundant in fruits, vegetables, and whole grains, include vitamins C and E, polyphenols, and carotenoids. Observational studies suggest that diets rich in antioxidants are associated with a reduced risk of certain cancers, such as colorectal and breast cancers. However, the efficacy of antioxidant supplements remains controversial as randomized controlled trials have yielded mixed results. In some cases, high doses of antioxidants have been shown to act as pro-oxidants, exacerbating oxidative stress rather than alleviating it. This discrepancy highlights the need for further research to elucidate the complex interactions between antioxidants, dietary components, and oxidative stress in cancer prevention [29,30].

Targeting oxidative stress represents a promising avenue for cancer prevention and therapy. By reducing ROS levels and enhancing antioxidant defenses, it may be possible to mitigate the DNA damage and genomic instability that drive tumor initiation. Moreover, antioxidants may improve the efficacy of cancer treatments by protecting normal cells from oxidative damage caused by chemotherapy and radiation therapy. However, given the ability of cancer cells to adapt to oxidative stress, therapeutic strategies must be carefully tailored to exploit the vulnerabilities of tumor cells while preserving normal tissue function. Future research should focus on identifying the optimal balance of pro-oxidants and antioxidants, as well as understanding the specific roles of oxidative stress in different cancer types, to develop more effective and personalized approaches to cancer prevention and management [31,32].

### 2.3. Insulin and IGF-1 Signaling

The insulin and insulin-like growth factor-1 (IGF-1) signaling pathways are central regulators of cellular metabolism, growth, and survival. Under normal physiological conditions, insulin facilitates glucose uptake and metabolism, while IGF-1 promotes cell growth and repair. However, dysregulation of these pathways, often influenced by diet and lifestyle factors, has been strongly implicated in cancer development and progression. Insulin and IGF-1 exert their effects by binding to their respective receptors, the insulin receptor (IR) and IGF-1 receptor (IGF-1R), which activate downstream signaling cascades. The two primary pathways activated are the phosphoinositide 3-kinase (PI3K)/Akt/mammalian target of rapamycin (mTOR) pathway and the Ras/mitogen-activated protein kinase (MAPK) pathway. These pathways promote cell proliferation, survival, and angiogenesis while inhibiting apoptosis, creating a microenvironment conducive to tumorigenesis [33,34].

Hyperactivation of these signaling pathways is often observed in cancer and is strongly influenced by dietary patterns. High-calorie diets, particularly those rich in refined carbohydrates and saturated fats, lead to hyperinsulinemia and elevated circulating IGF-1 levels. Hyperinsulinemia reduces the levels of insulin-like growth factor-binding proteins (IGFBPs), which normally sequester IGF-1, thereby increasing the bioavailability of IGF-1. This results in enhanced activation of IGF-1R and its downstream signaling pathways, promoting oncogenic processes such as cell proliferation and metastasis. Epidemiological studies have consistently demonstrated that elevated IGF-1 levels are associated with an increased risk of several cancers, including breast, colorectal, and prostate cancers [35,36].

Dietary interventions, on the other hand, offer promising strategies to modulate insulin and IGF-1 signaling and reduce cancer risk. Calorie restriction (CR) and intermittent fasting (IF) have been shown to lower circulating insulin and IGF-1 levels, thereby attenuating the activation of oncogenic pathways. In animal models, CR not only reduces tumor incidence but also enhances the efficacy of cancer therapies by sensitizing tumor cells to treatment-induced stress while protecting normal cells. Similarly, plant-based diets rich in fiber improve insulin sensitivity and reduce hyperinsulinemia, further mitigating cancer risk [37,38].

Specific dietary components also play a role in modulating these pathways. Diets high in plant-based proteins, whole grains, and legumes have been associated with lower IGF-1 levels, while diets rich in animal proteins, particularly dairy, have been linked to elevated IGF-1 levels. For instance, studies have shown that dairy protein, especially from milk, increases circulating IGF-1 levels, which may partly explain its association with prostate cancer risk. Conversely, omega-3 fatty acids, found in fatty fish and flaxseeds, exhibit anti-inflammatory and insulin-sensitizing effects, counteracting the pro-cancer effects of hyperinsulinemia and IGF-1 signaling [30,39].

The Western diet, characterized by high intakes of red meat, processed foods, and refined sugars, exacerbates insulin and IGF-1 signaling, promoting tumor growth and progression. In contrast, adherence to plant-based or Mediterranean dietary patterns, which emphasize fruits, vegetables, whole grains, and healthy fats, has been associated with reduced cancer risk. These diets not only improve metabolic health but also modulate the tumor microenvironment by reducing inflammation and oxidative stress, which are closely linked to insulin and IGF-1 signaling [38,40].

In conclusion, insulin and IGF-1 signaling pathways are critical drivers of cancer development and progression, and their dysregulation is strongly influenced by diet and nutrition. Dietary strategies such as calorie restriction, intermittent fasting, and plant-based diets offer effective means to modulate these pathways and reduce cancer risk. Future research should focus on personalized dietary interventions that consider individual metabolic profiles and genetic predispositions to optimize cancer prevention and management.

### 2.4. Cell Cycle Regulation

The cell cycle governs cellular growth, DNA replication, and division, ensuring genomic stability through a regulated network of cyclins, cyclin-dependent kinases (CDKs), and their inhibitors. This regulation prevents uncontrolled cell proliferation, and its dysregulation is a hallmark of cancer, allowing tumor cells to bypass checkpoints, evade apoptosis, and proliferate uncontrollably [41].

The cell cycle is divided into four phases: G1 (growth), S (DNA synthesis), G2 (preparation for mitosis), and M (mitosis). Progression through these phases is controlled by checkpoints. The G1/S checkpoint ensures DNA integrity, while the G2/M checkpoint verifies DNA replication accuracy. Cyclins and CDKs, such as cyclin D-CDK4/6 and cyclin E-CDK2, drive these transitions. Tumor suppressors like p53 and retinoblastoma protein (Rb) act as gatekeepers, halting the cycle in response to DNA damage. In cancer, mutations in these regulators—such as loss of p53 or CDK hyperactivation—disrupt cell cycle control, enabling unchecked proliferation [42,43].

Diet and nutrition significantly influence cell cycle regulation. Polyphenols like epigallocatechin gallate (EGCG) from green tea, curcumin from turmeric, and resveratrol from grapes inhibit cyclin D1 and CDK4/6, inducing cell cycle arrest in the G1 phase. Resveratrol also activates p53 and downregulates cyclin E-CDK2, arresting cells in the S phase. These compounds act through mechanisms such as epigenetic modifications and PI3K/Akt/mTOR pathway inhibition [44,45,46].

Calorie restriction and intermittent fasting reduce insulin and IGF-1 levels, downregulating pathways that drive cell cycle progression. These interventions promote cell cycle arrest and sensitize cancer cells to therapy, while protecting normal cells. Preclinical studies show that CR suppresses cyclins and CDKs while upregulating CDK inhibitors like p21 and p27, restoring cell cycle control [47,48].

Conversely, diets high in refined carbohydrates, saturated fats, and processed foods exacerbate cell cycle dysregulation by promoting hyperinsulinemia and chronic inflammation. Elevated insulin and IGF-1 activate the PI3K/Akt/mTOR pathway, driving unchecked cell proliferation. Chronic inflammation generates reactive oxygen species, damaging DNA, and further disrupting cell cycle regulation [39,49].

Micronutrients also play a role. Folate, found in leafy greens, is essential for DNA synthesis and repair, while selenium enhances p53 activity, promoting cell cycle arrest. Excessive iron intake, however, can induce oxidative stress, emphasizing the need for balanced nutrition [50,51].

In conclusion, cell cycle regulation is critical for genomic stability, and its dysregulation drives cancer progression. Dietary strategies such as CR, IF, and polyphenol-rich foods can restore cell cycle control and reduce cancer risk, offering promising avenues for personalized nutrition-based cancer prevention and therapy.

### 2.5. Apoptosis

Apoptosis, or programmed cell death, is a vital process for maintaining tissue homeostasis by eliminating damaged or harmful cells. It is characterized by cell shrinkage, chromatin condensation, and DNA fragmentation. Dysregulation of apoptosis is a hallmark of cancer, enabling tumor cells to evade death, accumulate mutations, and resist therapy [52,53].

Apoptosis occurs via two main pathways: the intrinsic (mitochondrial) pathway and the extrinsic (death receptor) pathway. The intrinsic pathway is regulated by the Bcl-2 family of proteins, where pro-apoptotic members like Bax promote mitochondrial outer membrane permeabilization, leading to caspase activation and cell death. The extrinsic pathway is triggered by external signals through receptors like Fas, activating caspase-8. Dysregulation of these pathways, such as overexpression of anti-apoptotic proteins (e.g., Bcl-2) or loss of pro-apoptotic regulators (e.g., p53), allows cancer cells to evade apoptosis and resist treatments [54,55].

Dietary components can restore apoptotic control and reduce cancer risk. Polyphenols like epigallocatechin gallate from green tea, curcumin from turmeric, and resveratrol from grapes modulate apoptosis by activating pro-apoptotic proteins (e.g., Bax) and inhibiting anti-apoptotic proteins (e.g., Bcl-2). These compounds also enhance caspase activation, inducing cell death in cancer cells [56,57]. Flavonoids such as quercetin (found in apples) and genistein (from soy) further promote apoptosis by modulating mitochondrial function and inhibiting survival pathways like PI3K/Akt [58].

Calorie restriction and intermittent fasting are dietary interventions that enhance apoptosis by reducing insulin and IGF-1 levels, thereby suppressing the PI3K/Akt/mTOR pathway. These interventions sensitize cancer cells to apoptotic signals while protecting normal cells, improving therapy outcomes [47,48]. Conversely, Western diets high in refined carbohydrates and fats promote chronic inflammation and oxidative stress, which inhibit apoptosis and increase cancer risk [59].

In summary, apoptosis is crucial for preventing cancer by eliminating abnormal cells. Dysregulation of apoptotic pathways allows tumor cells to thrive and resist treatment. Dietary strategies, including polyphenol-rich foods, flavonoids, and fasting regimens, offer promising approaches to restoring apoptotic control and reducing cancer risk.

## 3. Current Dietary Strategies

The established relationship between obesity and an increased risk of cancer, coupled with the metabolic demands of malignant cells for fuels such as glucose, amino acids, and fats, highlights the potential of dietary interventions in cancer prevention and therapy [60,61]. Obesity contributes to carcinogenesis through mechanisms such as chronic inflammation, insulin resistance, and elevated levels of growth factors like IGF-1 and leptin, which promote tumor growth and progression [62]. Additionally, cancer cells exhibit metabolic reprogramming, characterized by increased uptake and utilization of glucose, amino acids, and lipids to support their rapid proliferation and survival [13].

Given these insights, various dietary strategies have been developed to target cancer-related pathways, including calorie restriction, intermittent fasting, ketogenic diets, plant-based diets, and anti-inflammatory diets. These approaches aim to modulate processes such as cell cycle regulation, apoptosis, and inflammation, offering potential benefits in both cancer prevention and as adjuncts to conventional therapies [7,63]. However, each strategy has its own advantages, limitations, and contraindications, necessitating a personalized approach tailored to individual patient profiles and tumor characteristics.

Table 2 provides a comprehensive comparison of these dietary strategies, summarizing their mechanisms of action, benefits, and potential risks. This framework underscores the importance of integrating dietary interventions into cancer care while considering the unique metabolic and clinical needs of each patient [64].

### 3.1. Starvation and Caloric Restriction

Starvation and caloric restriction are dietary strategies that exploit the metabolic vulnerabilities of cancer cells, offering potential benefits in both cancer prevention and therapy. These approaches work by limiting the availability of metabolic fuels, such as glucose, amino acids, and fats, which are essential for the rapid proliferation of malignant cells. Numerous preclinical and clinical studies have demonstrated that CR and starvation can inhibit tumor growth, enhance the efficacy of conventional cancer therapies, and improve overall patient outcomes [47,48].

**Figure 1:**
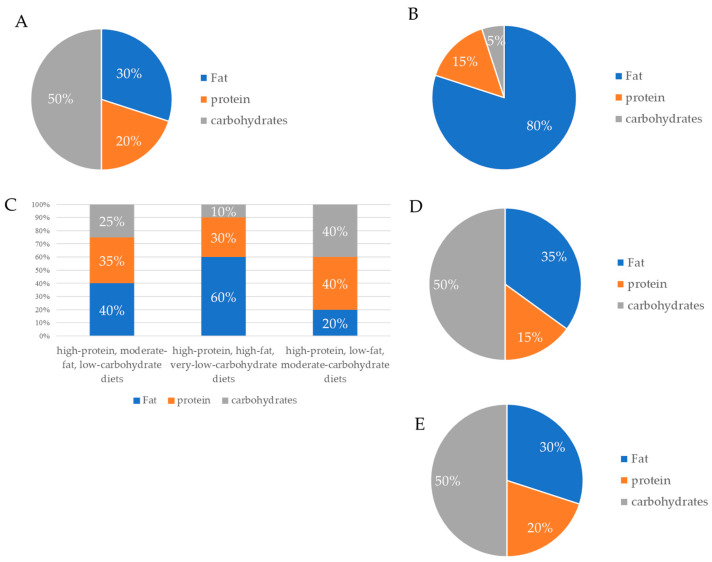
The macronutrient ratio: (**A**) caloric restriction diets; (**B**) ketogenic diet; (**C**) high-protein diets; (**D**) the Mediterranean diet; (**E**) vegetarian and vegan diets.

The typical composition of a caloric restriction diet includes approximately 55–65% carbohydrates, 30% fat, and 20% protein (Figure 1A), with the total caloric intake reduced by 20–40% compared to standard dietary recommendations [65]. This composition ensures that essential nutrients are maintained while reducing overall energy intake to create a systemic metabolic shift unfavorable to cancer cells.

Mechanistically, CR exerts its anticancer effects by downregulating key growth-promoting pathways, such as the insulin/IGF-1 and PI3K/Akt/mTOR signaling cascades. These pathways are critical for cellular growth and survival, and their suppression leads to reduced cell proliferation, enhanced autophagy, and increased apoptosis in tumor cells [66,67]. Additionally, CR reduces oxidative stress and inflammation, both of which are implicated in carcinogenesis. By decreasing the production of reactive oxygen species and pro-inflammatory cytokines, CR creates a less favorable microenvironment for tumor growth [68].

CR has also been shown to enhance the efficacy of chemotherapy and radiation therapy. Short-term fasting (STF), a form of CR, sensitizes cancer cells to treatment by inducing metabolic stress while simultaneously protecting normal cells. This differential effect is attributed to the reliance of cancer cells on glycolysis and their inability to adapt to nutrient deprivation, unlike normal cells [69,70]. Clinical trials have begun to explore the safety and efficacy of CR and fasting-mimicking diets (FMDs) in cancer patients, with preliminary results suggesting improved treatment outcomes and reduced therapy-related side effects [71,72].

Despite its promise, CR is not without challenges. Prolonged caloric restriction may lead to malnutrition, fatigue, and reduced quality of life, particularly in cancer patients with advanced disease or cachexia. Therefore, these strategies require careful monitoring and should be tailored to individual patient needs. Emerging approaches, such as intermittent fasting and FMDs, aim to replicate the metabolic effects of CR while minimizing the risks associated with prolonged nutrient deprivation [73,74].

In conclusion, starvation and caloric restriction represent innovative approaches to cancer therapy by leveraging the metabolic vulnerabilities of tumor cells. While these strategies hold significant potential, their application must be personalized and closely monitored to ensure patient safety and efficacy. Future research should focus on optimizing CR protocols and integrating them into comprehensive cancer care to maximize their therapeutic benefits.

### 3.2. The Ketogenic Diet

The ketogenic diet (KD) is a high-fat, low-carbohydrate, and moderate-protein dietary approach that has gained attention as a potential adjunctive therapy in cancer treatment. By inducing a metabolic state of ketosis, where ketone bodies are utilized as an alternative energy source, the KD creates an unfavorable environment for cancer cells, which are heavily dependent on glucose for energy production due to the Warburg effect [75]. This metabolic shift selectively starves cancer cells while preserving normal cell function, making KD a promising strategy for targeting tumor metabolism.

The standard composition of a ketogenic diet includes approximately 80–85% fat, 10–15% protein, and 5% carbohydrates, with a typical fat-to-carbohydrate ratio of 4:1 (Figure 1B) [76]. This macronutrient distribution minimizes glucose availability and promotes the production of ketone bodies, which most cancer cells cannot efficiently utilize due to their impaired mitochondrial function.

Mechanistically, the ketogenic diet exerts its anticancer effects through multiple pathways. By reducing circulating glucose and insulin levels, KD suppresses the insulin/IGF-1 signaling axis, a key driver of cancer cell proliferation and survival [68]. Additionally, the diet enhances oxidative stress within cancer cells by increasing the production of reactive oxygen species while depriving them of glucose, their primary energy source [77]. This combination of metabolic stress and nutrient deprivation promotes apoptosis and inhibits tumor progression.

The ketogenic diet also modulates the tumor microenvironment by reducing inflammation and angiogenesis, both of which are critical for tumor growth and metastasis [78,79]. Furthermore, preclinical studies have demonstrated that KD enhances the efficacy of chemotherapy, radiation therapy, and immunotherapy by sensitizing cancer cells to treatment while protecting normal cells from damage [80].

Despite its potential, clinical evidence for the ketogenic diet in cancer therapy remains limited and mixed. For instance, a clinical trial involving patients with glioblastoma multiforme reported that KD was well tolerated and associated with prolonged progression-free survival in some patients, but larger, randomized studies are needed to validate these findings [65,81]. Similarly, in pancreatic cancer patients undergoing chemoradiation therapy, KD improved treatment tolerability and reduced therapy-related side effects, although its impact on overall survival remains unclear [82].

However, the ketogenic diet is not without challenges. Common side effects include lethargy, nausea, and gastrointestinal discomfort, which can affect adherence, particularly in cancer patients undergoing intensive treatment [81]. Moreover, some studies have raised concerns about the potential for KD to promote tumor metastasis in specific contexts, highlighting the need for personalized dietary strategies [83].

In conclusion, the ketogenic diet represents a promising approach to cancer therapy by exploiting the metabolic vulnerabilities of tumor cells. However, its application must be tailored to individual patients based on tumor type, metabolic profile, and treatment goals. Future research should focus on identifying biomarkers to predict which patients are most likely to benefit from KD and optimizing its integration with standard cancer therapies.

### 3.3. High-Protein Diet

High-protein diets, characterized by a higher proportion of protein relative to fats and carbohydrates, exist in various forms (Figure 1C). Common variations include high-protein, moderate-fat, low-carbohydrate diets (approximately 30–40% protein, 30–40% fat, 20–30% carbohydrates); high-protein, high-fat, very-low-carbohydrate diets, resembling the ketogenic approach (approximately 30% protein, 60% fat, 10% carbohydrates); and less common high-protein, low-fat, moderate-carbohydrate diets (approximately 40% protein, 20% fat, 40% carbohydrates) [84]. This macronutrient distribution ensures an adequate supply of protein to support muscle repair and immune function while maintaining a balanced energy intake. This dietary approach has been extensively studied for its potential to preserve lean body mass, improve treatment outcomes, and support recovery during cancer therapy. However, its role in cancer prevention and management remains controversial, with evidence suggesting both potential benefits and risks depending on the source, quantity, and timing of protein intake [85].

High-protein diets offer several benefits for cancer patients, particularly those undergoing intensive treatments such as chemotherapy or radiation therapy. Protein plays a critical role in maintaining muscle mass, repairing tissues, and supporting immune function, all of which are essential for recovery and resilience during treatment. Clinical studies have demonstrated that high-protein diets can mitigate weight loss, improve muscle strength, and reduce hospitalization rates in cancer patients [86,87]. Additionally, protein supplementation has been associated with improved quality of life and functional outcomes, particularly in patients at risk for cancer cachexia, a condition characterized by severe muscle wasting and weight loss [88].

Mechanistically, protein intake stimulates muscle protein synthesis through the activation of the mTOR signaling pathway, helping preserve lean body mass and counteract the catabolic effects of cancer and its treatments. Furthermore, protein-rich diets may modulate immune responses, enhancing the body’s ability to fight infections and recover from treatment-related side effects [89]. However, excessive protein intake, particularly from animal sources, has been linked to elevated levels of insulin-like growth factor-1, which may promote tumor growth and progression in certain cancers, such as prostate and colorectal cancer [90,91].

The source of protein also plays a crucial role in determining the overall impact of the diet. While plant-based proteins are associated with lower cancer risks and improved metabolic health, animal-based proteins, particularly processed meats, have been linked to increased cancer incidence and mortality [7]. Additionally, high-protein diets that are low in carbohydrates may alter gut microbiota composition, reducing the production of cancer-protective metabolites and potentially increasing the risk of gastrointestinal complications [92].

Despite these concerns, high-protein diets remain a valuable tool in clinical settings to address the nutritional needs of cancer patients. For example, patients with gastrointestinal cancers or those undergoing chemoradiation therapy have shown improved outcomes with high-protein supplementation, including better treatment tolerability and reduced side effects [86]. However, careful monitoring is required to balance the benefits of protein intake with the potential risks of overconsumption, particularly in patients with pre-existing metabolic conditions or advanced-stage cancer.

In conclusion, the high-protein diet offers significant potential for supporting cancer patients by preserving lean body mass, enhancing recovery, and improving treatment tolerance. However, its implementation must be carefully tailored, considering the source and quantity of protein, as well as the patient’s overall metabolic and clinical profile. Future research should focus on optimizing protein intake and identifying patient subgroups that are most likely to benefit from this dietary approach.

### 3.4. The Mediterranean Diet

The Mediterranean diet (MD) is a plant-based dietary pattern inspired by the traditional eating habits of countries bordering the Mediterranean Sea. It emphasizes whole, minimally processed foods such as fruits, vegetables, whole grains, legumes, nuts, seeds, and olive oil, complemented by moderate consumption of fish, poultry, and dairy, and limited intake of red meat and sweets. This dietary approach is widely recognized for its health benefits, particularly in reducing the risk of chronic diseases, including cancer [93].

The macronutrient composition of the Mediterranean diet typically includes 45–55% carbohydrates, 30–35% fat (with a high proportion of monounsaturated fats from olive oil), and 15–20% protein (Figure 1D) [94]. This balanced distribution provides sufficient energy while promoting metabolic health, making it an ideal dietary model for cancer prevention and management.

The anticancer effects of the Mediterranean diet are attributed to its high content of bioactive compounds, including fiber, polyphenols, antioxidants, and omega-3 fatty acids. These components work synergistically to reduce oxidative stress, inflammation, and insulin resistance, all of which are implicated in carcinogenesis. For example, polyphenols found in olive oil and red wine exhibit antitumor properties by modulating cell signaling pathways and inducing apoptosis in cancer cells [95]. Similarly, omega-3 fatty acids from fish and nuts inhibit angiogenesis and metastasis, further suppressing tumor progression [96].

Clinical studies have consistently demonstrated the protective effects of the Mediterranean diet against various cancers. High adherence to this dietary pattern has been associated with a reduced risk of breast, colorectal, and prostate cancers, as well as improved survival rates among cancer patients [97,98]. For instance, a cohort study found that individuals who closely followed the Mediterranean diet had a 17% lower risk of cancer-related mortality compared to those with low adherence [99].

In addition to its preventive benefits, the Mediterranean diet has shown promise as an adjunctive therapy for cancer patients. Its anti-inflammatory and antioxidant properties may enhance the efficacy of conventional treatments, reduce therapy-related side effects, and improve overall quality of life. For example, cancer survivors adhering to the Mediterranean diet have reported lower levels of fatigue and better physical functioning during recovery [100].

Despite its numerous benefits, the Mediterranean diet is not without challenges. Adherence may be difficult for some individuals due to cultural or economic factors, and the diet’s relatively high fat content, primarily from olive oil and nuts, may lead to weight gain if consumed in excess. However, when implemented thoughtfully, the Mediterranean diet offers a sustainable and effective approach to cancer prevention and survivorship.

In conclusion, the Mediterranean diet represents a gold standard for healthy eating, with robust evidence supporting its role in reducing cancer risk and improving outcomes for cancer patients. Future research should focus on tailoring this dietary pattern to individual needs and exploring its potential synergistic effects with emerging cancer therapies.

### 3.5. Vegetarian and Vegan Diets

Vegetarian and vegan diets are plant-based dietary patterns that have gained significant attention for their potential role in cancer prevention and management. Vegetarian diets exclude meat but may include dairy and eggs, while vegan diets eliminate all animal-derived products. Both diets emphasize the consumption of fruits, vegetables, whole grains, legumes, nuts, and seeds, which are rich in fiber, antioxidants, and phytochemicals. These components are believed to contribute to the anticancer effects of plant-based diets [101,102].

The typical macronutrient composition of vegetarian and vegan diets includes 50–60% carbohydrates, 20–30% fat, and 15–20% protein, depending on the specific dietary choices and sources of plant-based foods (Figure 1E) [103]. This distribution provides a high intake of complex carbohydrates and dietary fiber, with lower levels of saturated fat compared to omnivorous diets.

Vegetarian and vegan diets are associated with several potential benefits for cancer prevention. Studies have shown that these diets are linked to a modest reduction in overall cancer risk, with vegetarian diets reducing cancer incidence by 10–12% in some large-scale observational studies [104]. The high intake of plant-based foods provides a rich source of antioxidants and phytochemicals, which help neutralize free radicals, reduce oxidative stress, and modulate inflammation—key factors in carcinogenesis [105]. Additionally, the fiber content in these diets promotes gut health and supports the production of short-chain fatty acids, which have been shown to have anticancer properties [106].

In terms of cancer survivorship, vegetarian and vegan diets may improve treatment outcomes and enhance quality of life. Higher adherence to plant-based diets has been associated with better prognosis in cancer survivors, particularly for colorectal and prostate cancers [107]. These diets may also help mitigate treatment-related side effects, such as fatigue and inflammation, by providing a nutrient-dense and anti-inflammatory dietary profile [108].

However, vegetarian and vegan diets are not without challenges. One of the primary concerns is the potential for nutrient deficiencies, particularly in protein, vitamin B12, iron, zinc, and omega-3 fatty acids, which are more abundant in animal-based foods. For example, vegan diets are often lower in protein intake compared to other dietary patterns, which may require careful planning to ensure adequate consumption of plant-based protein sources such as legumes, tofu, tempeh, and quinoa [109]. Supplementation with vitamin B12 and fortified foods is also recommended to prevent deficiencies that could compromise immune function and overall health [110].

Another consideration is the reliance on processed plant-based foods, which can be high in added sugars, unhealthy fats, and sodium. These products may undermine the health benefits of a plant-based diet if consumed excessively. Therefore, a focus on whole, minimally processed plant foods is essential to maximize the anticancer potential of vegetarian and vegan diets [104].

In conclusion, vegetarian and vegan diets offer significant promise for cancer prevention and survivorship by emphasizing nutrient-dense, plant-based foods that reduce inflammation and oxidative stress. However, careful dietary planning and supplementation are necessary to address potential nutrient deficiencies and ensure a balanced intake of macronutrients and micronutrients. Future research should explore the long-term effects of these diets on cancer outcomes and identify strategies to optimize their implementation in diverse populations.

## 4. Diet and the Gut Microbiota

The gut microbiota, a complex community of microorganisms inhabiting the gastrointestinal tract, plays a central role in regulating human health. It influences metabolic processes, immune function, and inflammation, all of which are critical in cancer development, progression, and treatment outcomes. Diet is one of the most powerful modulators of the gut microbiota, with specific dietary patterns shaping microbial diversity and activity in ways that can either promote or reduce cancer risk [111,112].

Dietary fiber, a key component of plant-based diets, is a primary substrate for microbial fermentation in the colon. This fermentation process produces short-chain fatty acids (SCFAs), including butyrate, acetate, and propionate, which have profound anti-inflammatory and anticancer effects. Butyrate, in particular, acts as a histone deacetylase (HDAC) inhibitor, regulating gene expression, promoting apoptosis in cancer cells, and enhancing the differentiation of regulatory T cells (Tregs). These effects collectively strengthen the intestinal barrier, reduce inflammation, and support immune surveillance, thereby lowering cancer risk [112,113].

Conversely, diets high in saturated fats, refined sugars, and processed foods—hallmarks of the Western diet—are associated with gut dysbiosis. Dysbiosis refers to an imbalance in the microbial community, characterized by reduced microbial diversity and an overgrowth of pathogenic bacteria. This imbalance leads to the production of pro-inflammatory metabolites, such as secondary bile acids and lipopolysaccharides (LPS), which can damage the intestinal lining, promote chronic inflammation, and increase the likelihood of DNA damage. These mechanisms are strongly implicated in the development of colorectal cancer (CRC) [114,115].

The Mediterranean diet, which emphasizes fruits, vegetables, olive oil, nuts, and fish, is a prime example of a dietary pattern that supports a healthy gut microbiota. It promotes the growth of beneficial bacteria such as Faecalibacterium prausnitzii and Roseburia, which are known for their anti-inflammatory effects. Adherence to the Mediterranean diet has been linked to a lower risk of CRC and improved outcomes in cancer patients [112,113].

Similarly, vegetarian and vegan diets, which are high in fiber and low in saturated fat, encourage the proliferation of SCFA-producing bacteria and reduce the abundance of pathogenic species. These plant-based diets have been associated with lower risks of CRC and other cancers, likely due to their positive effects on gut health [116]. On the other hand, the ketogenic diet, which is high in fat and low in carbohydrates, induces significant changes in the gut microbiota. KD reduces the abundance of carbohydrate-fermenting bacteria while increasing the levels of Akkermansia muciniphila, a bacterium associated with improved metabolic health and enhanced responses to immunotherapy in cancer patients. However, long-term adherence to KD may reduce microbial diversity, raising concerns about its implications for gut health [116,117].

The gut microbiota also plays a pivotal role in modulating the efficacy and toxicity of cancer therapies, including chemotherapy, radiation therapy, and immunotherapy. Certain bacterial species enhance the effectiveness of treatments by stimulating antitumor immune responses. For instance, Akkermansia muciniphila has been linked to improved responses to immune checkpoint inhibitors. Conversely, an imbalanced microbiota can impair treatment outcomes and increase side effects, such as gastrointestinal toxicity during chemotherapy or radiation therapy [113,118,119].

Dietary interventions targeting the gut microbiota are emerging as promising strategies to improve cancer outcomes. Prebiotics, which are nondigestible fibers that feed beneficial bacteria, and probiotics, which are live beneficial microbes, have shown potential in restoring microbial balance, reducing inflammation, and enhancing the efficacy of cancer treatments. Additionally, fecal microbiota transplantation (FMT), though still experimental in cancer care, is being explored as a way to modulate the gut microbiota and improve therapeutic responses [120,121].

Despite these advances, challenges remain. The gut microbiota varies significantly between individuals, influenced by factors such as genetics, age, and lifestyle. This variability makes it difficult to establish universal dietary recommendations. Furthermore, the long-term effects of specific diets on the microbiota and cancer outcomes require further investigation through large-scale studies. Advances in metagenomics and metabolomics will enable a deeper understanding of the complex interactions between diet, gut microbiota, and cancer, paving the way for targeted dietary interventions to complement traditional cancer treatments.

In conclusion, diet is a powerful modulator of the gut microbiota, influencing cancer risk, progression, and treatment outcomes. While dietary patterns such as the Mediterranean and plant-based diets promote a healthy gut microbiota and reduce cancer risk, Western diets are associated with dysbiosis and increased cancer susceptibility. Understanding the interplay between diet, gut microbiota, and cancer will enable the development of personalized dietary interventions to optimize cancer prevention and therapy.

## 5. Conclusions

The intricate relationship between diet, cancer development, and progression underscores the critical role of nutrition in modern oncology. This review highlights the profound influence of dietary patterns and specific nutrients on molecular mechanisms central to carcinogenesis, including inflammation, oxidative stress, insulin signaling, cell cycle regulation, and apoptosis. Furthermore, the gut microbiota emerges as a pivotal mediator in the diet–cancer interplay, modulating immune responses and therapeutic outcomes.

While current evidence strongly supports the potential of dietary interventions in cancer prevention and treatment, significant challenges remain. Specifically, translating this knowledge into effective personalized dietary recommendations requires a more nuanced understanding of the variability in individual responses to dietary strategies. Factors such as tumor type, stage of disease, genetic predisposition, and metabolic profile must be considered to develop truly personalized dietary recommendations. Additionally, the long-term safety and efficacy of dietary interventions, such as caloric restriction, ketogenic diets, and plant-based approaches, require further validation through large-scale, randomized clinical trials.

Key insights from this review include the following:

1. Targeting Tumor Metabolism: Dietary interventions such as caloric restriction and ketogenic diets can exploit the metabolic vulnerabilities of cancer cells, potentially enhancing the efficacy of conventional therapies.
2. Modulating Inflammation and Oxidative Stress: Diets rich in antioxidants, polyphenols, and omega-3 fatty acids can mitigate chronic inflammation and oxidative stress, key drivers of tumorigenesis.
3. Gut Microbiota as a Therapeutic Target: The gut microbiota plays a critical role in shaping cancer risk and treatment responses. Dietary patterns that promote microbial diversity, such as the Mediterranean and plant-based diets, offer promising avenues for cancer prevention and management.
4. Personalized Nutrition: The integration of dietary strategies tailored to the molecular and clinical characteristics of individual patients holds immense potential for improving outcomes and quality of life.

Future research should prioritize the development of precision nutrition frameworks that incorporate biomarkers of dietary response, microbiome profiles, and tumor-specific metabolic dependencies. By bridging the gap between molecular oncology and nutritional science, we can pave the way for innovative, patient-centered approaches to cancer prevention and care.

In conclusion, diet is not merely a modifiable risk factor but a powerful tool in the fight against cancer. The integration of personalized dietary interventions into comprehensive cancer care has the potential to revolutionize prevention and treatment strategies, ultimately improving patient outcomes and quality of life.

## Figures and Tables

**Table 1:** Oncogenesis and nutrition interactions.

Mechanism	Description	Nutritional Factors Involved
Inflammation	Chronic inflammation promotes tumor development and progression in various types of cancer.	- Omega-3 fatty acids (anti-inflammatory) - Refined carbohydrates and saturated fats (pro-inflammatory)
Oxidative stress	Imbalance between pro-oxidants and antioxidants leads to DNA damage and mutations, contributing to carcinogenesis.	- Antioxidants (e.g., vitamins C and E, polyphenols) - Prooxidants (e.g., heterocyclic amines in charred meat)
Insulin and IGF-1 signaling	Overactivation of insulin and IGF-1 pathways promotes cell proliferation and survival in multiple cancer types.	- High glycemic load diets (increase insulin and IGF-1) - Calorie restriction (decrease insulin and IGF-1)
Cell cycle regulation	Dysregulation of cell cycle checkpoints leads to uncontrolled cell division, a hallmark of cancer.	- Folate and vitamin B12 (essential for DNA synthesis and repair) - Phytochemicals (e.g., curcumin, resveratrol)
Apoptosis	Evasion of programmed cell death allows cancer cells to survive and proliferate.	- Omega-3 fatty acids and flavonoids (induce apoptosis) - Saturated fats (inhibit apoptosis)

**Table 2:** Current dietary strategies.

Diet Type	Definition
Calorie restriction	Reducing total calorie intake over an extended period, lasting from a few months to several years.
Starvation	Starvation refers to an acute shortage of calories over an extended period of time, resulting in exhaustion. Most fasting methods allow unrestricted access to water, which is why they are also called water-only fasting.
Intermittent fasting	Short-term weekly fasting of 24 h, once or twice a week.
Ketogenic diet	A diet high in fat, low in carbohydrates, and low or high in protein intake.
High-protein diet	A diet in which >20% of calories come from protein.
Mediterranean diet	A diet based on the traditional diet and habits of the Mediterranean countries of Portugal, Spain, Italy, and Greece. It includes a high consumption of fruits, vegetables, and legumes, and moderate consumption of unprocessed cereals, olive oil, fish, and dairy products, with occasional consumption of meat and wine.
Vegetarian and vegan diets	The diet includes a high intake of fruits, vegetables, legumes, and unprocessed grains, with or without dairy products (lactovegetarianism) (veganism). The diet excludes meat, seafood, and poultry. Some varieties of vegetarian diets also include eggs (ovo-lacto-vegetarianism), fish (pescetarianism), or both (ovo-pescetarianism).

## Data Availability

No new data were created or analyzed in this study.
